# Experience of Using a Smartphone Mood Relapse Warning Application among Patients with Bipolar Disorders: A Qualitative Inquiry

**DOI:** 10.1192/j.eurpsy.2022.1460

**Published:** 2022-09-01

**Authors:** E.C.-L. Lin, M.-S. Chiou

**Affiliations:** 1 National Cheng Kung University, Taiwan, Department Of Nursing, Tainan City, Taiwan; 2 National Cheng Kung University, Taiwan, Department Of Nursing, Tainan, Taiwan

**Keywords:** Smartphone, bipolar disorder, Relapse Warning, Qualitative study

## Abstract

**Introduction:**

Although several studies preliminary supported the effects of using smartphone mental health application (app) in patients with bipolar disorder (BD), patients’ subjective experience deserves more attention.

**Objectives:**

The present study aimed to explore how the BD patient experienced while using the APP in detecting their mood relapse warning signs (MRW app) which has been developed by our team (Su et al., 2021).

**Methods:**

The MRW app collects 2 passive (location and GPS removal distance) and 6 self-reported data (daily mood, wake and sleep time, the brief record of mood and life, voice pitch, speech tone and rhythm, facial expression, and weekly emotional scale). By using qualitative research design, 15 patients recruited from the psychiatric outpatient department in a medical center were in-depth interviewed.

**Results:**

Four themes were identified as their subjective experience to use the app as: including positive and negative experience, facilitators, price, and barriers. Interconnected relationship was found in each theme; and counterbalancing associations between positive vs. negative experience, facilitators vs. price and barriers were also demonstrated.

**Conclusions:**

Such first-person experience of using the app in illness detection could unveil technological myths and present its impacts upon patients’ lives in the real world. Implication for practice and future studies were be discussed.

**Disclosure:**

No significant relationships.

